# Supplementary material for: Unveiling the Bioactive Potential of the Invasive Jellyfish Phyllorhiza punctata Through Integrative Transcriptomic and Proteomic Analyses
Source: Biomolecules. 2025 Aug 4;15(8):1121. doi: 10.3390/biom15081121 (PMC12383608; doi:10.3390/biom15081121)
Supplement: Supplementary file 1 [file biomolecules-15-01121-s001.zip › Suplementary Material/FileS3/PEPFOLD4/XP_065058978_15472/clusters.html]

 PEP-FOLD clusters


Download archive of all models  here   

|  |  |  |
| --- | --- | --- |
| # | file | sOPEP |
| # cluster 1 - Size: 99 |  |  |
|  | XP\_065058978\_15472-model1 | -21.7218 |
|  | XP\_065058978\_15472-model1.1 | -21.6535 |
|  | XP\_065058978\_15472-model1.2 | -21.5876 |
|  | XP\_065058978\_15472-model1.3 | -21.5299 |
|  | XP\_065058978\_15472-model1.4 | -21.5299 |
|  | XP\_065058978\_15472-model1.5 | -21.5267 |
|  | XP\_065058978\_15472-model1.6 | -21.5043 |
|  | XP\_065058978\_15472-model1.7 | -21.4882 |
|  | XP\_065058978\_15472-model1.8 | -21.4591 |
|  | XP\_065058978\_15472-model1.9 | -21.4591 |
|  | XP\_065058978\_15472-model1.10 | -21.4591 |
|  | XP\_065058978\_15472-model1.11 | -21.4591 |
|  | XP\_065058978\_15472-model1.12 | -21.4467 |
|  | XP\_065058978\_15472-model1.13 | -21.427 |
|  | XP\_065058978\_15472-model1.14 | -21.3634 |
|  | XP\_065058978\_15472-model1.15 | -21.3511 |
|  | XP\_065058978\_15472-model1.16 | -21.3438 |
|  | XP\_065058978\_15472-model1.17 | -21.3361 |
|  | XP\_065058978\_15472-model1.18 | -21.3238 |
|  | XP\_065058978\_15472-model1.19 | -21.3194 |
|  | XP\_065058978\_15472-model1.20 | -21.3189 |
|  | XP\_065058978\_15472-model1.21 | -21.2992 |
|  | XP\_065058978\_15472-model1.22 | -21.2992 |
|  | XP\_065058978\_15472-model1.23 | -21.2953 |
|  | XP\_065058978\_15472-model1.24 | -21.2842 |
|  | XP\_065058978\_15472-model1.25 | -21.2838 |
|  | XP\_065058978\_15472-model1.26 | -21.2809 |
|  | XP\_065058978\_15472-model1.27 | -21.275 |
|  | XP\_065058978\_15472-model1.28 | -21.2703 |
|  | XP\_065058978\_15472-model1.29 | -21.2676 |
|  | XP\_065058978\_15472-model1.30 | -21.2644 |
|  | XP\_065058978\_15472-model1.31 | -21.2564 |
|  | XP\_065058978\_15472-model1.32 | -21.2403 |
|  | XP\_065058978\_15472-model1.33 | -21.2222 |
|  | XP\_065058978\_15472-model1.34 | -21.2131 |
|  | XP\_065058978\_15472-model1.35 | -21.2087 |
|  | XP\_065058978\_15472-model1.36 | -21.2087 |
|  | XP\_065058978\_15472-model1.37 | -21.2049 |
|  | XP\_065058978\_15472-model1.38 | -21.2048 |
|  | XP\_065058978\_15472-model1.39 | -21.196 |
|  | XP\_065058978\_15472-model1.40 | -21.1945 |
|  | XP\_065058978\_15472-model1.41 | -21.1859 |
|  | XP\_065058978\_15472-model1.42 | -21.1859 |
|  | XP\_065058978\_15472-model1.43 | -21.1845 |
|  | XP\_065058978\_15472-model1.44 | -21.1809 |
|  | XP\_065058978\_15472-model1.45 | -21.1809 |
|  | XP\_065058978\_15472-model1.46 | -21.1809 |
|  | XP\_065058978\_15472-model1.47 | -21.1702 |
|  | XP\_065058978\_15472-model1.48 | -21.1671 |
|  | XP\_065058978\_15472-model1.49 | -21.1629 |
|  | XP\_065058978\_15472-model1.50 | -21.1591 |
|  | XP\_065058978\_15472-model1.51 | -21.1543 |
|  | XP\_065058978\_15472-model1.52 | -21.1408 |
|  | XP\_065058978\_15472-model1.53 | -21.1408 |
|  | XP\_065058978\_15472-model1.54 | -21.1365 |
|  | XP\_065058978\_15472-model1.55 | -21.1362 |
|  | XP\_065058978\_15472-model1.56 | -21.1313 |
|  | XP\_065058978\_15472-model1.57 | -21.1256 |
|  | XP\_065058978\_15472-model1.58 | -21.1196 |
|  | XP\_065058978\_15472-model1.59 | -21.1178 |
|  | XP\_065058978\_15472-model1.60 | -21.1178 |
|  | XP\_065058978\_15472-model1.61 | -21.1154 |
|  | XP\_065058978\_15472-model1.62 | -21.1119 |
|  | XP\_065058978\_15472-model1.63 | -21.1047 |
|  | XP\_065058978\_15472-model1.64 | -21.0975 |
|  | XP\_065058978\_15472-model1.65 | -21.0975 |
|  | XP\_065058978\_15472-model1.66 | -21.0923 |
|  | XP\_065058978\_15472-model1.67 | -21.0913 |
|  | XP\_065058978\_15472-model1.68 | -21.07 |
|  | XP\_065058978\_15472-model1.69 | -21.0636 |
|  | XP\_065058978\_15472-model1.70 | -21.0633 |
|  | XP\_065058978\_15472-model1.71 | -21.0424 |
|  | XP\_065058978\_15472-model1.72 | -21.0424 |
|  | XP\_065058978\_15472-model1.73 | -21.042 |
|  | XP\_065058978\_15472-model1.74 | -21.0389 |
|  | XP\_065058978\_15472-model1.75 | -21.0305 |
|  | XP\_065058978\_15472-model1.76 | -21.0296 |
|  | XP\_065058978\_15472-model1.77 | -21.0296 |
|  | XP\_065058978\_15472-model1.78 | -21.0264 |
|  | XP\_065058978\_15472-model1.79 | -21.0232 |
|  | XP\_065058978\_15472-model1.80 | -21.022 |
|  | XP\_065058978\_15472-model1.81 | -21.022 |
|  | XP\_065058978\_15472-model1.82 | -21.0204 |
|  | XP\_065058978\_15472-model1.83 | -21.0191 |
|  | XP\_065058978\_15472-model1.84 | -21.0178 |
|  | XP\_065058978\_15472-model1.85 | -21.0152 |
|  | XP\_065058978\_15472-model1.86 | -21.0145 |
|  | XP\_065058978\_15472-model1.87 | -21.0047 |
|  | XP\_065058978\_15472-model1.88 | -20.999 |
|  | XP\_065058978\_15472-model1.89 | -20.9784 |
|  | XP\_065058978\_15472-model1.90 | -20.9665 |
|  | XP\_065058978\_15472-model1.91 | -20.9639 |
|  | XP\_065058978\_15472-model1.92 | -20.9629 |
|  | XP\_065058978\_15472-model1.93 | -20.921 |
|  | XP\_065058978\_15472-model1.94 | -20.8753 |
|  | XP\_065058978\_15472-model1.95 | -20.87 |
|  | XP\_065058978\_15472-model1.96 | -20.8673 |
|  | XP\_065058978\_15472-model1.97 | -20.8478 |
|  | XP\_065058978\_15472-model1.98 | -20.7976 |
| # cluster 2 - Size: 1 |  |  |
|  | XP\_065058978\_15472-model2 | -17.839 |
|  |  |  |
